# Autologous organoid co-culture model reveals T cell-driven epithelial cell death in Crohn’s Disease

**DOI:** 10.3389/fimmu.2022.1008456

**Published:** 2022-11-10

**Authors:** Nassim Hammoudi, Sarah Hamoudi, Julie Bonnereau, Hugo Bottois, Kevin Pérez, Madeleine Bezault, Déborah Hassid, Victor Chardiny, Céline Grand, Brice Gergaud, Joëlle Bonnet, Leila Chedouba, My-Linh Tran Minh, Jean-Marc Gornet, Clotilde Baudry, Hélène Corte, Léon Maggiori, Antoine Toubert, Jacqueline McBride, Camille Brochier, Margaret Neighbors, Lionel Le Bourhis, Matthieu Allez

**Affiliations:** ^1^ Université de Paris, INSERM U1160, EMiLy, Institut de Recherche Saint-Louis, Paris, France; ^2^ Gastroenterology Department, AP-HP, Hôpital Saint-Louis/Lariboisière, Paris, France; ^3^ Digestive Surgery Department, AP-HP, Hôpital Saint-Louis/Lariboisière, Paris, France; ^4^ OMNI Biomarker Development, Genentech Inc., South San Francisco, CA, United States; ^5^ Ile de France, Institut Roche, Boulogne-Billancourt, France

**Keywords:** lymphoepithelial interactions, crohn’s disease, inflammatory bowel diseases, organoids, CD103, NKG2D

## Abstract

Lympho-epithelial interactions between intestinal T resident memory cells (Trm) and the epithelium have been associated with inflammatory bowel disease (IBD) activity. We developed ex vivo autologous organoid-mucosal T cell cocultures to functionally assess lymphoepithelial interactions in Crohn’s Disease (CD) patients compared to controls. We demonstrate the direct epithelial cell death induced by autologous mucosal T cells in CD patients but not in controls. These findings were positively correlated with T cell infiltration of the organoids. This potential was inhibited by limiting lympho-epithelial interactions through CD103 and NKG2D blocking antibodies. These data directly demonstrate for the first time the direct deleterious effect of mucosal T cells on the epithelium of CD patients. Such ex-vivo models are promising techniques to unravel the pathophysiology of these diseases and the potential mode of action of current and future therapies.

## Introduction

The large number of T lymphocytes present in the ileal mucosa, their important diversity and their interrelation with the rest of the immune system, the microbiota and the intestinal barrier make them major players in intestinal homeostasis with a definite role in the pathophysiology of Inflammatory Bowel Diseases (IBD) (1).

Resident memory T (Trm) cells, present in mucosal tissues close to the potential site of arrival of pathogens have distinct transcriptional and phenotypic signatures compared to blood memory T cells (2–4). Phenotypically, they constitute a heterogeneous population with various functions. Their involvement in the pathophysiology of IBD remains controversial. Previous studies from Lloyd Mayer’s group reported altered interactions between T lymphocytes and epithelial cells in IBD (5–8). Several receptors expressed at the surface of mucosal T lymphocytes are involved in direct interaction with epithelial cells, including CD103 (the alphaE-beta7 integrin) and a number of activating receptors such as NKG2D.

CD103 is a major phenotypic marker of tissue resident memory cells (9, 10) which might play an important role in intestinal homeostasis. This molecule is formed by the alphaE and beta7 subunits that bind to E-Cadherin expressed by epithelial cells. Its expression is higher on CD8 than CD4 T cells, especially in the small intestine (11, 12). We recently demonstrated that CD8 T cells expressing CD103 in Crohn’s Disease (CD) can induce a tissue-wide alert increasing innate immune responses and recruitment of effector cells *via* the production of cytokines of the Th17 pathway (13). In addition, the presence of CD4 T cells expressing CD69 and CD103 cells in the mucosa of IBD patients was demonstrated to be predictive of the development of flares (10).

NKG2D is a cell receptor that plays a role in intestinal immune homeostasis and inflammation, expressed at the surface of all CD8 T and NK cells in humans. The ligands are MHC class I chain-related proteins A and B (MICA, MICB) and UL1-6-binding proteins (ULBPs 1-6), expressed at basal levels on intestinal epithelial cells and upregulated under stress conditions (14). The engagement of this receptor leads to activation and production of pro-inflammatory cytokines (15). In celiac disease, CD8 T intraepithelial lymphocytes have been shown to be cytotoxic over duodenal epithelial cells through NKG2D engagement independently of the TCR (16–18). We showed an increased rate of CD4 T lymphocytes expressing NKG2D in the lamina propria of CD patients. Activation of these cells *via* TCR stimulation associated with NKG2D-MICA binding induced cytotoxicity towards epithelial cells and the production of pro-inflammatory cytokines such as INFg, TNFalpha and IL17 (19–21). We and others also described the upregulation of several NKG2D ligands on the epithelium in IBD (19, 22).

One major breakthrough in the field of cell biology in the past decade is the so-called “organoid” technology that consists of the culture of epithelial stem cells embedded in extracellular matrix forming multicellular three dimensional structures (23, 24). Two recent studies showed that organoids derived from active lesions of IBD patients maintained permanent changes in mRNA expression in the stem cell compartment (25, 26). These transcriptional modifications were also associated with altered functions of the epithelium.

On the other hand, T cells may have a direct or indirect (through the production of cytokines) effect on epithelial cells. A recent study underlined the close and interdependent interactions between the immune system and the epithelial cells showing that they may orchestrate tissue-wide responses to external signals (27). This is particularly true in inflammatory conditions where intestinal stem cells at the bottom of the crypts can upregulate class II major histocompatibility complex (MHC) molecules predominantly at the basolateral membrane secondary to an increased IFNgamma secretion by both innate and adaptive immune cells. Furthermore, a recent study elegantly demonstrated that IFNgamma production by T cells under inflammatory conditions could have a pro-apoptotic effect on epithelial stem cells (28). Taken together, these studies underscore the need to study lymphoepithelial interactions and their consequences in organoid autologous cultures from IBD patients.

Herein, we developed an innovative ex vivo autologous co-culture model to analyze the impact of mucosal T lymphocytes on epithelial cells in CD. We showed that T cells from patients with CD have an increased cytotoxic potential over autologous epithelial cells compared to control conditions. We then demonstrated that the cell receptors NKG2D and CD103 had an important role in this process as blocking antibodies abolished the cytotoxic responses. These data were in line with the differential expression of these receptors on mucosal T cells in two cohorts of patients according to intestinal inflammatory status.

## Results

### Ileal mucosal T cells from patients with CD have an increased cytotoxic potential over Caco2 spheroids

**Figure 1:**
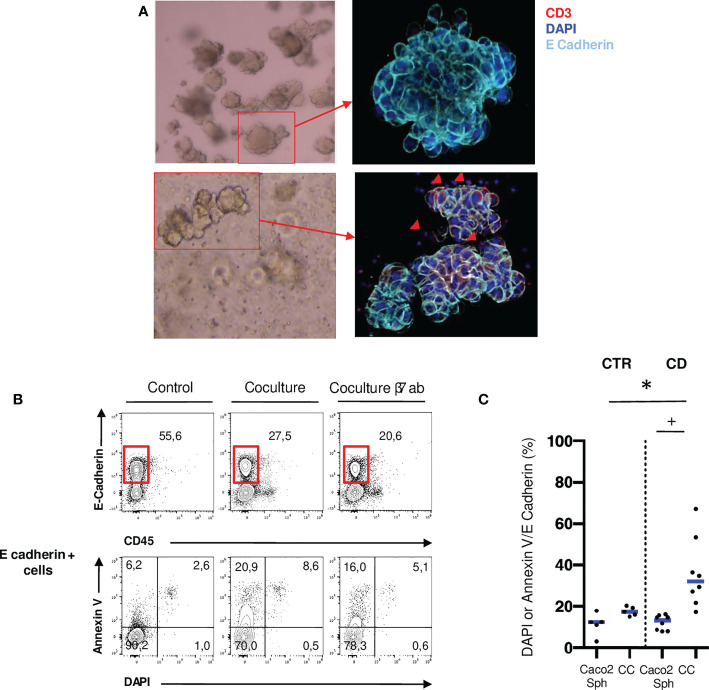
Ileal mucosal T cells from patients with active CD have an increased cytotoxic potential over Caco2 spheroids. **(A)** Caco2 spheroids alone or in coculture with mucosal T cells from CD patients in brightfield microscopy (10X) and confocal microscopy (200X: Blue: DAPI; Light Blue: E-Cadherin). Red arrows correspond to T cells infiltrating the spheroid (Red: CD3). **(B)** Gating strategy to assess Caco2 cell death with or without T cells in cocultures. Cell death was assessed through Annexin V and DAPI staining in E-Cadherin positive cells. **(C)** Comparison of Caco2 cells death rates between allogenic cocultures with mucosal T cells from controls and active CD patients. CD= Crohn’s Disease, CTR= Control. CTR: n=5; CD: n=8. *p < 0.05 Mann Whitney Test. +: p < 0.05 Wilcoxon paired Test.

To investigate the functional role of mucosal T cells populations, we performed allogenic co-cultures between ileal mucosal lymphocytes isolated from 8 CD patients and 5 controls and Caco2 spheroids (**Figure 1A**). These immortalized cell lines express NKG2D ligands and E-Cadherin and appropriately cultured in matrigel, they form 3D structures resembling organoids (29, 30). Epithelial cell death was assessed through DAPI and Annexin V staining by flow cytometry (**Figure 1B**). Epithelial cell death was significantly increased in co-cultures with CD mucosal lymphocytes but not with control lymphocytes (**Figure 1C**). These results show that ileal mucosal lymphocytes from CD patients have an increased cytotoxic potential on epithelial cells in an allogenic model compared to control ileal mucosal lymphocytes.

### Autologous co-cultures reveal direct interactions between mucosal lymphocytes and epithelial organoids especially in CD patients

**Figure 2:**
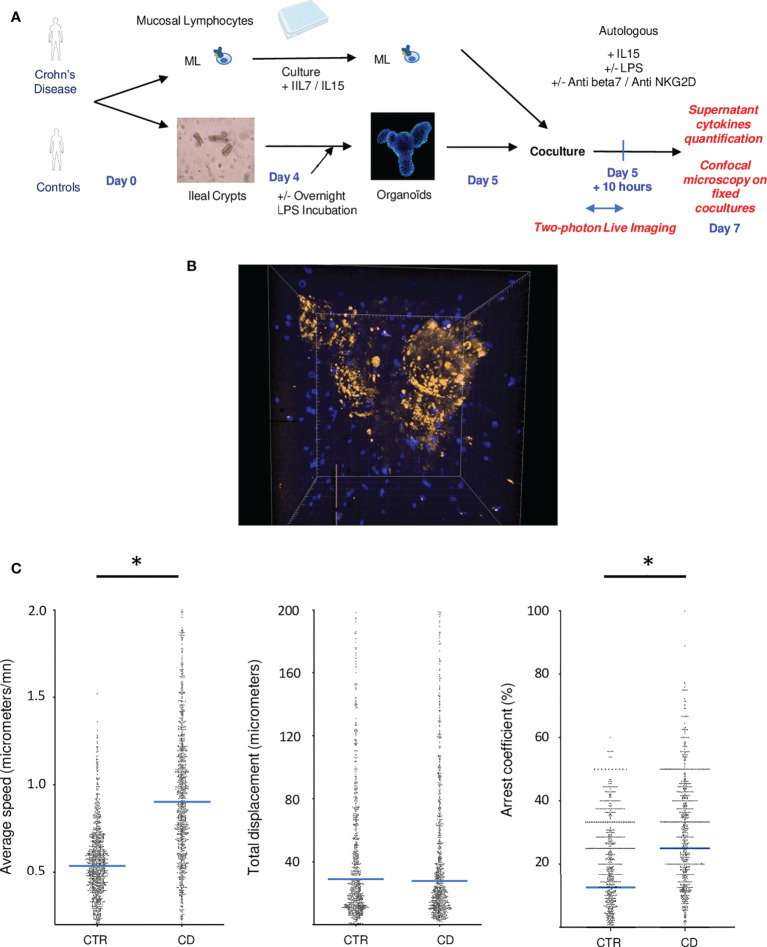
Autologous co-cultures reveal direct interactions between mucosal lymphocytes and epithelial organoids especially in CD patients. **(A)** Experimental design of cocultures **(B)** Example of a two photon acquisition for a CD patient showing multiple interactions between T cells and an organoid. **(C)** Average speed, total displacement and arrest coefficient of lymphocytes in cocultures. Each dot corresponds to a lymphocyte. Cells from different individuals were combined in this figure. These figures represent 2 control patients and 3 IBD patients. CD= Crohn’s Disease, CTR= Control. *p < 0.05 Mann Whitney Test.

Allogenic responses are extremely variable by nature and cannot reliably recapitulate lymphoepithelial interactions, hence we developed organoid autologous co-cultures. Co-culture between organoids and mucosal lymphocytes from the same patient was performed 5 days after crypts and immune cell extractions (**Figure 2A**). To visualize the interactions of mucosal lymphocytes with organoids in cocultures, we performed 2-photon microscopy during the first ten hours of coculture (**Figure 2B**; **Video 1**). It revealed the capacity of mucosal T cells to come in close contact and infiltrate organoids (**Figure 2B**).

T cell tracking quantification revealed the increase activation of mucosal lymphocytes in CD experiments. Indeed, T cells presented a higher average speed and arrest coefficient, compared to healthy controls (**Figure 2C**), suggesting that T cells formed prolonged and more numerous interactions with organoids in this condition.

### Autologous co-cultures reveal increased mucosal T cell activation against epithelial cells in CD patients

**Figure 3:**
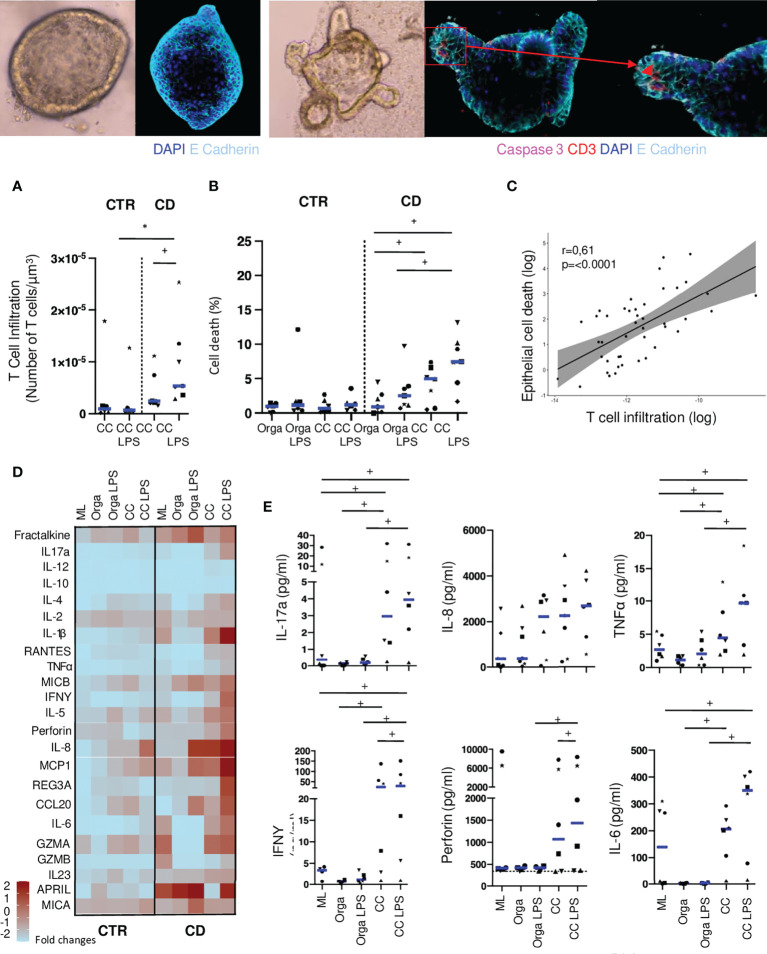
Autologous T cells from CD patients infiltrate organoids and induce epithelial cell death. **(A)** T cell infiltration in the organoids evaluated by the number of CD3 positive-cells in contact or infiltrating each organoid with or without LPS preincubation. Each dot corresponds to the median of the values obtained for organoids of a given patient. Each shape of point corresponds to a given patient. CTR: n=6, CD: n=7. **(B)** Organoid epithelial cell death evaluated by Caspase 3 fluorescence area over organoid volume in organoids alone or cocultures with or without LPS preincubation. CTR: n=6, CD: n=7 **(C)** Correlation between T cell infiltration and epithelial cell death in coculture with LPS preincubation. Each dot corresponds to the absolute value obtained for an organoid. **(D)** Cytokine detection matrix in the supernatants. Each square represents the median value of the results obtained for each well of a given condition in the distinct patients. The color of each square stands for the level expression. CTR: n=6 CD: n=6. **(E)** Absolute values of cytokine quantification of 6 analytes (IL-17a, IL-8, TNFalpha, IFNgamma, Perforin and IL-6) in the supernatants from CD patients. Each dot corresponds to the absolute value obtained for a given condition. Each shape of point corresponds to a given patient. CC= Coculture, CD= Crohn’s Disease, CTR= Control, LPS= Lipopolysaccharide ML= Mucosal Lymphocytes, Orga= Organoid. +: p < 0.05 Wilcoxon paired Test, *p < 0.05 Mann-Whitney Test.

We then quantified these lymphoepithelial interactions in autologous co-cultures in presence or absence of LPS. We observed that T cells from CD patients tended to infiltrate more autologous organoids than with cells from control ileum (**Figure 3A**). This difference reached statistical significance in conditions with LPS, mimicking the addition of a bacterial trigger, (**Figure 3A**), suggesting that a basal level of inflammation is participating to this process.

In the absence of lymphocytes, the level of epithelial cell apoptosis was not different between organoids from controls or CD patients, with or without LPS (**Figure 3B**). Co-cultures with autologous lymphocytes did not increase epithelial cell apoptosis in control patients. However, with cells from CD patients, epithelial cell death was significantly higher when adding LPS in the co-cultures (**Figure 3B**; **Supplementary Figure 1**). Concomitantly, in CD experiments but not in controls, organoid volume in co-culture with LPS was significantly reduced as compared to organoids alone or in co-culture without LPS (**Supplementary Figures 2A, B**). Interestingly, in co-cultures from CD patients with LPS incubation, T cell infiltration of the organoid was positively correlated with epithelial cell death (**Figure 3C**). These results suggest the cytotoxic potential of autologous T cells against epithelial cells modulated by microbial cues in CD patients.

We then compared the secretion of soluble factors, such as cytokines and chemokines, under these conditions (**Figure 3D**). Across patients, production of cytokines were highly variable (**Figure 3E**). Organoids from CD patients were more prone to release chemokines and cytokines (i.e. IL-8, Fractalkine, CCL20 or MCP1) as well as soluble stress molecules (MICA, MICB), which was further increased when adding LPS. In the supernatants from mucosal lymphocytes of CD patients incubated alone, we found higher concentrations of pro-inflammatory cytokines such as IL-17a, IL-1beta, TNFalpha, or IL-6 and of granzyme A and B (**Figures 3D, E**).

In co-culture experiments, organoids incubated with LPS released significantly higher levels of the chemokines CCL20 and Fractalkine as well as the soluble forms of MICB with cells from CD patients compared to controls (**Figure 3D**). This finding underlined the ability of organoids to respond to LPS stimulation, chemo-attract immune cells and eventually interact with them. IL-5, IL-6, IL-17a, TNFalpha and IFNgamma were also significantly increased in co-culture conditions with LPS as compared to both organoids and mucosal lymphocytes incubated alone. The cytolytic protein Perforin was detected in significantly higher concentrations in co-culture conditions with LPS than in co-cultures without, only in conditions with cells from CD patients, confirming a potential direct cytotoxic effect of T cells from CD patients, towards the organoids (**Figures 3D, E**).

### Monoclonal antibodies targeting lympho-epithelial interactions reduce epithelial cell death in autologous co-cultures

**Figure 4:**
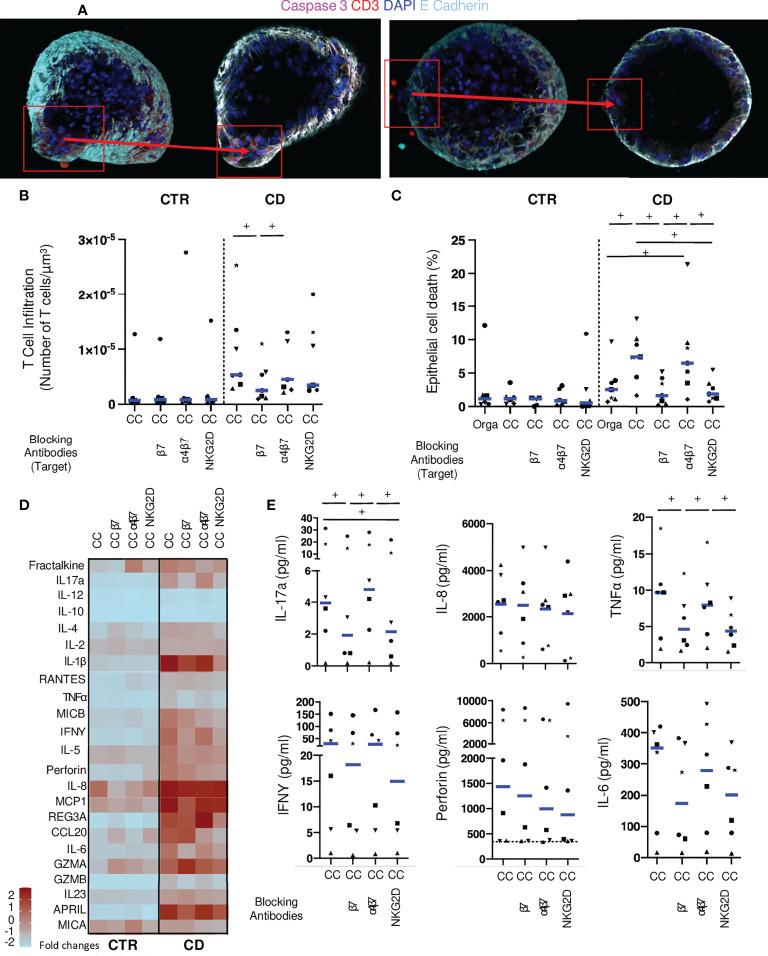
Monoclonal antibodies targeting lympho-epithelial interactions reduce epithelial cell death in co-cultures. **(A)** Organoid in coculture with mucosal T cells from CD patients without (left) or with anti-beta7 (right) in confocal microscopy (200X: Blue: DAPI; Light Blue: E-Cadherin, Red CD3, Pink: Caspase 3). **(B)** T cell infiltration in the organoids evaluated by the number of CD3 positive-cells in contact or infiltrating each organoid with LPS incubation. Each dot corresponds to the median of the values obtained for organoids of a given patient. Each shape of point corresponds to a given patient. CTR: n=6, CD: n=7. **(C)** Organoid epithelial cell death evaluated by Caspase 3 fluorescence area over organoid volume in organoids alone or cocultures with LPS incubation. CTR: n=6, CD: n=7. **(D)** Cytokine detection matrix in the supernatant in LPS coculture conditions. Each square represents the median value of the results obtained for each well of a given condition in the distinct patients. The color of each square stands for the level expression (Form light blue: low expression to deep red: high expression). CTR: n=6, CD: n=6. **(E)** Absolute values of cytokine quantification of 6 analytes (IL-17a, IL-8, TNFalpha, IFNgamma, Perforin and IL-6) for CD patients. Each dot corresponds to the absolute value obtained for a given condition (with LPS). Each shape of point corresponds to a given patient. CC= Coculture, CD= Crohn’s Disease, CTR= Control, ML= Mucosal Lymphocytes, Orga= Organoid. +: p < 0.05 Wilcoxon paired test.

We then tested the potential involvement of proteins involved in direct contacts between epithelial and T cells, CD103 and NKG2D. We used antibodies targeting beta7 (Etrolizumab), that will block CD103; as well as an anti-alpha4-beta7 (vedolizumab) as a negative control. Additionally, antibodies against the NKG2D receptor on lymphocytes were added to the autologous cocultures (**Figure 4A**). We did not detect any effect on T cell infiltration for the blocking conditions in control co-cultures. In co-culture conditions without LPS, anti-beta7 significantly reduced CD patients’ derived T cell infiltration of autologous organoids compared to normal co-culture, while anti-alpha4-beta7 and anti-NKG2D had no effect (**Supplementary Figure 3A**). No effect on epithelial cell death was noted (**Supplementary Figure 3B**). These results show that blocking beta7 reduced T cells interactions with epithelial cells.

Under inflammatory conditions with LPS, anti-beta7 significantly reduced the number of T lymphocytes infiltrating the organoid in experiments with cells from CD patients but not controls (**Figure 4B**). No impact on T cell infiltration was found in presence of anti-alpha4-beta7 and anti-NKG2D antibodies. Importantly, both anti-beta7 and anti-NKG2D, but not anti-alpha4-beta7, significantly reduced epithelial cell death, in these conditions (**Figure 4C**). These results strongly suggest that T cells derived from CD patients but not controls have a direct cytotoxic effect, through CD103 and NKG2D, on autologous epithelial cells in inflammatory conditions.

We then measured soluble factors in the supernatants of these inhibition experiments. No difference was found in cytokines levels when adding blocking conditions in control co-cultures (**Supplementary Figure 3C**). We found a significant reduction of IL-5, IL-6, TNFalpha, IL-17a and IFNgamma in co-cultures with anti-beta7 as compared to no blockade antibody in experiments with LPS. There was a significant reduction of IL-6 and IL-17a and a trend for reduction of IL-5, TNFalpha, and IL-1beta in presence of anti-NKG2D antibody. Additionally, MICB and CCL20 were significantly lower in conditions with the latter as compared to controls. Interestingly, a trend towards less perforin release was found with anti-NKG2D as compared to controls (**Figures 4D, E**). These results confirm the CD103 and NKG2D-dependent responses in conditions with CD patient-derived T cells against autologous organoids.

### CD103 and NKG2D on mucosal T cells are associated with IBD disease activity

**Figure 5:**
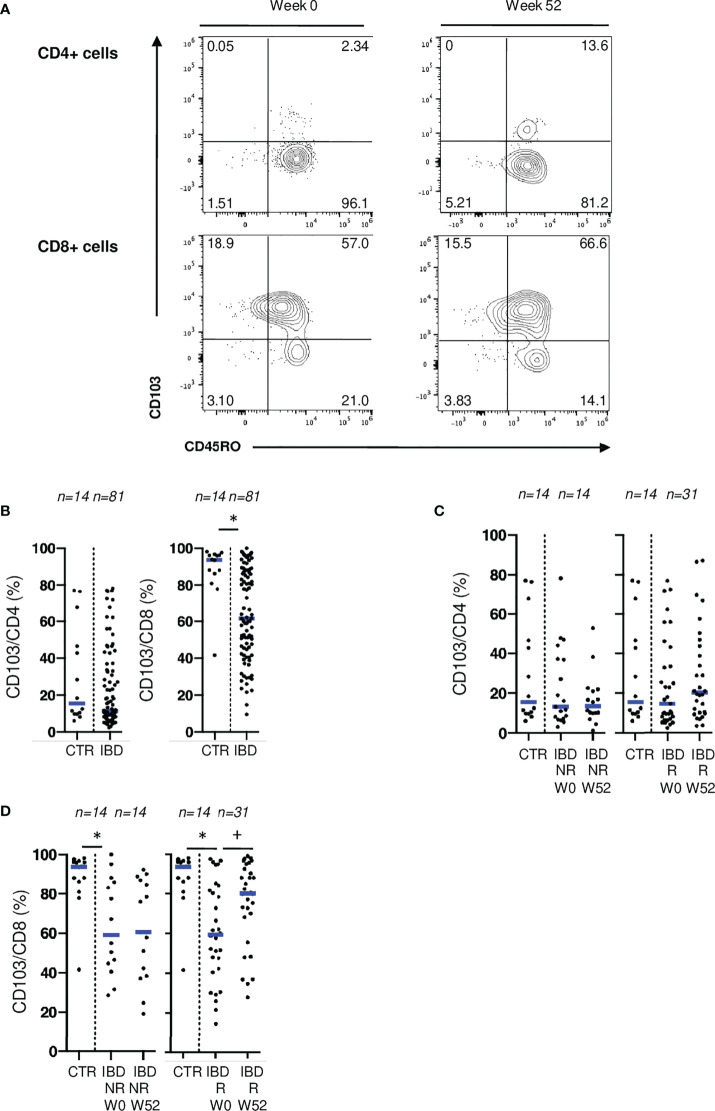
CD103 expression on mucosal T cells is associated with IBD disease activity. **(A)** Gating strategy for CD103 and CD45RO stainings in flow cytometry. **(B)** Comparison of mucosal rates of CD4 and CD8 T cells expressing CD103 between controls and patients from the ELYP cohort at week 0. **(C)** Evolution of mucosal rate of CD8 T cells expressing CD103 rates in IBD patients from the ELYP cohort without or with endoscopic response to biotherapy. **(D)** Evolution of mucosal rate of CD4 T cells expressing CD103 rates in IBD patients from the ELYP cohort without or with endoscopic response to biotherapy. CTR: ileal mucosal samples) CD= Crohn’s Disease, CTR= Control, IBD= Inflammatory Bowel Disease, NR= Non Responders, R= Responders. *p < 0.05 Mann Whitney Test; +: p < 0.05 Wilcoxon Paired Test.

To confirm the involvement of CD103 and NKG2D in disease activity, we analyzed the expression of these receptors, on mucosal T cells from patients undergoing medical or surgical treatments (**Supplementary Figure 4**). No difference was found when comparing blood CD4 and CD8 T cells expressing CD103 with controls (**Supplementary Figure 5A**). In mucosal T cells, there was a widespread expression of CD103 on CD4 and CD8 T cells. However, before biologic therapy initiation, IBD patients displayed a lower percentage of mucosal CD8 T cells expressing CD103 compared to control patients (**Figures 5A, B**). Endoscopic responders at W52 experienced an increase of this mucosal population back to non-IBD control levels (**Figure 5D**). In contrast, rates of mucosal CD8 T cells expressing CD103 were stable over time in non-responders (**Figure 5D**). Rates of CD4 T cells expressing CD103 were not different between IBD patients and controls both at W0 and W52 (**Figures 5B, C**). Similar results were found in the cohort of patients undergoing ileo-cecal surgical resection (**Supplementary Figure 5B**). These results suggest a specific and reversible alteration of both CD4 and CD8 Trm populations during inflammation especially involving CD103.

Expression of CD103 was positively and significantly correlated with CD45RO and CCR6 on mucosal CD4 T cells and with CD161, CCR6 and CD39 on CD8 T cells (**Supplementary Figure 6**), markers associated with Th17 lineage, hence underlining the pro-inflammatory potential of T cells expressing CD103 in IBD.

**Figure 6:**
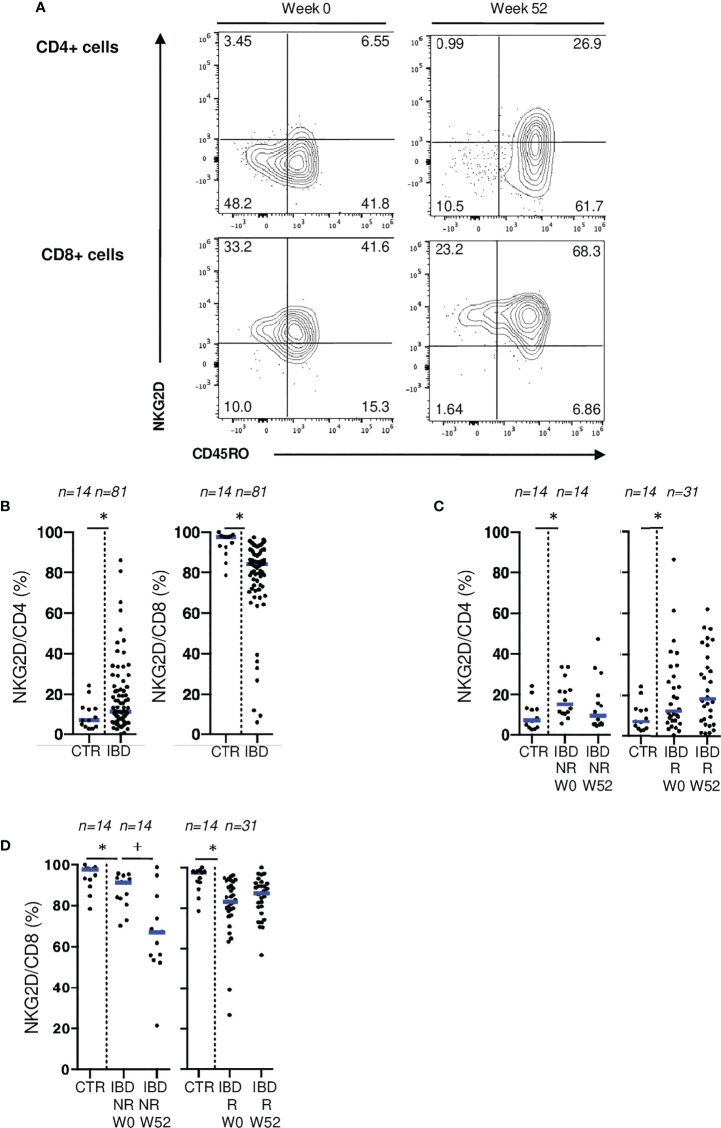
NKG2D expression on mucosal T cells is associated with IBD disease activity. **(A)** Gating strategy for CD45RO and NKG2D stainings in flow cytometry. **(B)** Comparison of mucosal rates of CD4 and CD8 T cells expressing NKG2D between controls and patients from the ELYP cohort at week 0. **(C)** Evolution of mucosal rate of CD8 T cells expressing NKG2D rates in IBD patients from the ELYP cohort without or with endoscopic response to biotherapy. **(D)** Evolution of mucosal rate of CD4 T cells expressing NKG2D rates in IBD patients from the ELYP cohort without or with endoscopic response to biotherapy. CD= Crohn’s Disease, CTR= Control, IBD= Inflammatory Bowel Disease, NR= Non Responders, R= Responders. *p < 0.05 Mann Whitney Test; +: p < 0.05 Wilcoxon Paired Test.

Similarly, no difference was found when comparing blood CD4 and CD8 T cells expressing NKG2D between control and CD patients (**Supplementary Figure 5D**). However, in patients with active IBD, we found a lower surface expression of NKG2D on mucosal CD8 T cells, and higher rates of mucosal CD4 T cells expressing NKG2D than controls (**Figures 6A, B**). Similar results were found in the surgical cohort (**Supplementary Figure 5C**). To further explore the downregulation of NKG2D on mucosal T cells in active IBD, we performed ex vivo stimulation of mucosal T cells from IBD patients through CD3 and NKG2D using coated beads. We demonstrated that mucosal T cell activation through NKG2D led to the downregulation of this receptor at cell surface reflecting its engagement. This downregulation of NKG2D on mucosal CD8 T cells isolated from IBD patients is hence probably due to active stimulation through this pathway (**Supplementary Figure 7**). Interestingly, NKG2D expression on mucosal CD8+ T cells decreased even further in endoscopic non-responders at W52, which was not the case in responders (**Figure 6D**). We also observed a non-significant increase of NKG2D expression on mucosal CD4+ T cells from responders while it remained low in non-responders (**Figure 6C**). Taken together, these results suggest that NKG2D engagement is associated with disease activity.

## Discussion

Using an innovative autologous co-culture model, we demonstrated the deleterious effect of mucosal T cells towards epithelial cells. Inhibition of lympho-epithelial interactions through NKG2D or CD103 blockade, abrogated this T cell-induced epithelial cell death. Phenotypic analyses of mucosal T cells in IBD patients also showed significant differences in the distribution of mucosal T cell subsets and highlighted the importance of the lympho-epithelial interactions receptors CD103 and NKG2D.

Among several surface markers analyzed in our cohorts on alpha/beta T cells, the receptors most significantly differentially expressed with inflammation were CD103 and NKG2D. Furthermore, these abnormal expressions of CD103 and NKG2D were reverted in responders to biotherapies but not in non-responders. For CD103 expression on CD8 T cells, comparable findings were made in two recent studies of patients with active IBD and during their follow-up (31, 32).

While recent works in oncology reported the development of allogeneic or autologous co-culture models to understand the mechanisms of tumor development, their resistance to immune responses and their sensitivity to treatments (33, 34), such approach have not yet been developed in IBD. In our experiments, the addition of LPS resulted in an increase in organoid death in co-cultures with mucosal lymphocytes from CD patients but not from controls. Epithelial cell death correlated with increased T cell infiltration, and perforin was significantly increased in the supernatants of co-cultures. Organoids from CD patients incubated with LPS showed an increased release of chemokines such as CCL20, Fractalkine or IL-8 and cell stress products such as MICB. Interestingly, a recently published work showed an increase permeability of intestinal epithelial cells under the effect of LPS associated with an increase in IL-8 production in cell lines (35).

Our results strongly suggest a direct cytotoxic effect of T cells on the epithelium. The effect observed could be related to CD8 T cells but also to cytotoxic CD4 T cells subpopulations, as previously described by our group (19). *In vivo*, different T cell subsets likely interact altogether, and inflammation may also result from an imbalance between these subsets. For these reasons, we think there is a benefit to perform these co-cultures experiments with all mucosal T cells. Further studies may decipher the impact of each subset in this process and potential synergies between them.

Inter-individual variation of cytokine productions was high reflecting that additional factors including patient heterogeneity or previous treatment exposure might play a role. Despite this wide spread, paired statistical analysis show that various pro-inflammatory cytokines such as IFNgamma, TNFalpha, IL-6 or more specifically IL-17a were found in increased concentrations in the supernatants of co-cultures from CD patients. These findings may suggest a combined Th1 and Th17 involvement in the deleterious effect of the immune cells in co-cultures. Interestingly anti-beta7 (Etrolizumab) and anti-NKG2D had comparable effects in terms of inhibition of cell death. However, anti-beta7 significantly reduced T cell infiltration and proinflammatory cytokines release (TNFalpha, IL-6, IFNgamma, IL-17a), which was not the case for anti-NKG2D, suggesting that these blocking antibodies exert their respective effect through different mechanisms.

Dai et al. recently show in murine models that blockade of the beta7 integrin reduced retention and increased egress of both lamina propria and intraepithelial T cells from the mucosa. Through the transcriptomic analysis of mucosal T cells from CD patients treated with etrolizumab, the authors also show that mucosal CD103 T cells are mainly pro-inflammatory with little to no regulatory markers and that beta7 blockade reduced mucosal inflammatory T cell gene expression including TNFA or IFNG (36). Our results are in line with these findings, going even further by demonstrating the epithelial consequences of this blockade in a human autologous ex vivo model.

In contrast to anti-beta7 (etrolizumab), anti-NKG2D antibody had no effect on T cell infiltration. Anti-NKG2D was associated with the reduction of markers of epithelial stress and a trend towards a reduction of perforin in the supernatants. These data were in line with both *in vitro* and flow cytometry data presented in the manuscript especially showing an engagement of the NKG2D receptor in active inflammatory lesions. All these findings suggest an implication of the NKG2D receptor in a direct cytotoxic effect of mucosal T cells towards epithelial cells. Anti-alpha4-beta7 was used as a negative control and no such effect was seen with this monoclonal antibody. This was expected given the absence of the ligand of this antibody (MADCAM) in our co-culture model. These data confirm the potential value of monoclonal antibody treatments targeting lymphoepithelial interactions (37, 38). However, while clinical trials assessing the efficacy of etrolizumab and anti-NKG2D showed sign of biological responses, the clinical results were negative, reflecting the complexity of intestinal mucosal immunology and the possibility that these treatments may be suitable for a subpopulation of IBD patients.

In our work, we considered mucosal lymphocytes, without separating intraepithelial from lamina propria lymphocytes. A recent single cell analysis of peripheral and mucosal lymphocytes from IBD patients indeed show phenotypic differences between these two lymphocyte subpopulations (39). However, a continuum and a largely shared TCR repertoire between T cells of the lamina propria and the epithelium has recently been demonstrated in both CD4 and CD8 T cells (40). Furthermore, the fixed view of the position of the cells given by anatomopathological studies of the digestive mucosa, is challenged by recent dynamic studies of lymphoepithelial interactions that highlight the continuous mobility of lymphocytes in contact with the epithelium and within the matrix (41). Finally, while phenotypic characteristics differentiate intra-epithelial and lamina propria lymphocytes, it is important to keep in mind the important plasticity of mucosal T cells and their capacity to acquire surface markers and functional specificities linked to the inflammatory and cytokine context (42, 43). From our point of view, in this approach, it seemed more legitimate to study the mucosal lymphocytes as tissue resident memory cells in their entirety.

There are several limitations to our study. Even if we identified the role of two specific receptors, we do not know yet which specific T cell subsets are involved in these pro-inflammatory and cytotoxic processes. The addition of IL-15 in our co-cultures to enhance lymphocyte survival could be a limitation of our work but it was also added in controls and no co-culture effects were found. Moreover, the expression of IL15 in the mucosa of patients with CD and UC as in celiac disease has been reported to be higher as compared to controls (44–46). We also reported that a particular subset of CD4 T cells expressing NKG2D displays a higher level of IL15-R in the active mucosal lesions of IBD patients (19). In this regard, it could be argued that the addition of IL15 could even reflect the situation within the mucosa of patients with this digestive diseases. Our experiments did not allow us to precisely understand the mechanisms underlying the autologous cell death, the precise phenotype or antigenic specificity of the T cells implicated. Due to the nature of the work carried out, a significant consumption of material was necessary, and a considerable number of organoids and lymphocytes had to be used, which does not allow the application of the model as presented in this work in clinical practice.

## Conclusion

These data demonstrate for the first time, to our knowledge, the direct deleterious effect of mucosal T cells on the epithelium of CD patients using a novel co-culture model. Lymphoepithelial interactions appear implicated in this process. Such ex-vivo models, based on organoids and autologous immune cells, could be used to identify new targets, to further understand mechanisms of actions of new biologics, and could be used to further explore the potential benefit of combination of therapies.

## Methods and materials

### Subject details

Human ileal samples were retrieved from patients included in three different cohorts:

The ELYP cohort, a prospective single-center study, included patients with IBD who have started a biotherapy for active disease (**Supplementary Table 1**). Mucosal biopsies in the most inflamed segment were taken during endoscopies before treatment initiation (Week 0: W0) and in the same location 52 weeks (W52) after inclusion. Control biopsies were taken from 16 patients without IBD (7 ileal and 9 colonic) (**Supplementary Figure 4A**).

The REMIND cohort included patients since September 2010. Inclusion criteria were: >18 years of age, ileal or ileocolonic CD and indication of CD-related intestinal surgery (ileocolonic resection) (**Supplementary Table 2**). All patients had, 6 months after surgery, a colonoscopy to assess the endoscopic recurrence according to the Rutgeerts score (**Supplementary Figure 4B**).

The IMCO cohort included prospectively since February 2017 patients who underwent resection for colon cancer at the Saint-Louis Hospital. For patients with right colon cancer, ileum was then available for analysis.

### Definition of endoscopic response to biotherapy

For ELYP patients, subsequent endoscopies were performed at W52 after biotherapy initiation. The Crohn’s Disease Endoscopic Index Score (CDEIS) and the Ulcerative Colitis Endoscopic Index Score (UCEIS) were respectively assessed at both endpoints for CD and UC patients. For Crohn’s Disease (CD), response was defined by a 50% decrease of the CDEIS score. For Ulcerative Colitis (UC), response was defined by a 2-point decrease of the UCEIS.

### Method details

#### Mucosal lymphocytes and peripheral blood mononuclear cells isolation

For PBMCs, blood was collected at each timepoints from patients of the 3 different cohorts. For MLs, surgical specimens were washed with PBS solution. The mucosa was stripped from the submucosa cut into small pieces and enzymatically and mechanically digested under rotation in a solution containing collagenase IV (Sigma-Aldrich) and DNase I (Roche). The supernatants were then filtered, and the mononuclear cells layered through a density gradient technique using Ficoll medium.

#### Flow Cytometry analysis of the sorted lymphocytes

MLs and PBMCs phenotypes from patients’ samples of the ELYP, REMIND and IMCO cohorts at different timepoints were performed on an Attune NxT Flow Cytometer (ThermoFisher). Acquired data were analyzed using FlowJo 10.4.2 software (TreeStar).

#### Antibodies used for flow cytometry experiments

MLs phenotypes from patients’ samples of the ELYP cohort at different timepoints were performed using CD103-FITC, CD73-PE, CTLA4-APC, CD39-APC-Vio770, CCR6-PE, PD1-PE-Vio770, NKG2D-APC, CD45RO-APC-Vio770 (Miltenyi), CD3-AF700, CD161-PE-Cy5, CD8-BV605 and CD4-BV711 (BD Biosciences), . MLs phenotypes from patients’ samples of the REMIND and IMCO cohort at different timepoints were performed using CD69-FITC, CD45RO-PerCP, CD5-AF700, CD8-BV605, CD4-BV711 (BD Biosciences), NKG2D-APC and CD103-APC-Vio770 (Miltenyi). All samples were co-stained with DAPI to assess cell viability and diluted in optimal concentration in FACS buffer (Miltenyi).

For the allogenic cocultures, cells were then stained using the following antibodies: Annexin V-FITC, E-Cadherin-PE-Vio770 HLA-E-APC, MICA/MICB-APC-Vio770 (Miltenyi), HLA-ABC-AF700 (BioLegend), HLA-DPDQDR-BV510, CD45-BV605 (BD Biosciences) and ULBP 2/5/6-PE (RD). All samples were co-stained with DAPI to assess cell viability and diluted in optimal concentration in Annexin V binding buffer (Miltenyi).

#### *In vitro* mucosal lymphocytes culture

MLs isolated were cultured in complete RPMI medium supplemented with IL-15 and IL-7 (Miltenyi).

#### Caco2 spheroids formation

Caco2 spheroids were generated by seeding 2500 Caco2 cells (ATCC (cat. HTB-37)) in 20 μl Matrigel matrix (Corning, 100%). After an incubation of 5 days polarized empty spheroids with an approximative diameter of 150 μm were formed and recuperated.

#### Crypt isolation, organoid culture and incubation with LPS

Surgical specimens from the healthy ileum of CD patients of patients with right colon cancer were used. Briefly, the mucosa was stripped from the submucosa cut into small pieces and incubated sequentially in an antibiotic solution, then in DL-Dithiothreitol (DTT) solution and in an Ethylene Diamine Tetra-acetic Acid (EDTA) solution at 4°C. Samples were then immerged in cold PBS and shaken vigorously to extract the intestinal crypts.

Crypts were cultivated in Matrigel (Corning) drops at the concentration of approximately 100 crypts per drop. Intesticult (STEM CELL Technologies), supplemented with Y-27632 (STEM CELL Technologies), was then added. Medium was first changed at 24 hours with Y-27632 free-Intesticult and then every two days.

After 5 days of crypt culture, organoids were formed and ready to be co-cultivated. The day before the planned co-culture selected wells were incubated overnight with a LPS solution at the concentration of 1 μg/ml.

#### Co-cultures

Co-culture was performed 5 days after initiation of Caco2 spheroid or crypt culture. In parallel, ileal MLs were cultured in complete RPMI medium supplemented with IL-15 and IL-7 also for 5 days. Ileal MLs, spheroids and organoids were retrieved from the culture wells, washed and pooled separately. Co-cultures were then performed at the ratio of 500 MLs per spheroid for allogenic co-cultures and 10000 MLs per organoid for the autologous co-cultures. Medium was then added supplemented with IL-15 at the concentration of 10 ng/ml, LPS at the concentration of 1 μg/ml if required and the desired blockade antibodies. Blockade antibodies used were etrolizumab (anti beta7 antibody at the concentration at 5 μg/ml– Roche/Genentech), vedolizumab as a control, (anti alpha4-beta7 antibody at the concentration of 5 μg/ml – Roche/Genentech) and anti-NKG2D (at the concentration of 5 μg/ml as previously described (34) ThermoFisher).

In parallel of the fixed coculture, when enough material was remaining, one part of the organoids and their autologous mucosal lymphocytes were, respectively stained with a far-red cell tracker (Invitrogen, 1/1000 dilution) and anti-CD45-Pacific blue antibody (Thermofisher, clone HI30) and a co-culture was made with the same ratios in Matrigel as autologous cocultures for two photon live acquisition. Acquisition was performed by two-photon microscope of the Saint Louis platform (LSM 780, Leica). Images were acquired every 5 minutes for 10 hours.

### Co-cultures endpoints assessment

#### Flow Cytometry analysis of the allogenic co-cultures

Allogenic co-cultures were recovered at 48 hours. Cells were analyzed on an Attune NxT Flow Cytometer (ThermoFisher). Acquired data were analyzed using FlowJo 10.4.2 software (TreeStar).

#### Cytokines quantification in the co-cultures experiments

Supernatants were retrieved 48 hours after co-culture initiation. Cytokine quantification was performed using the Luminex Technology (Luminex Corp). Invitrogen ProcartaPlex (ThermoFisher) were used and samples were processed according to the manufacturer instructions. Supernatants were processed on a MAGPIX instrument (Luminex Corp) and analyzed with the MAGPIX software.

#### Cytokines quantified in the supernatants of the autologous cocultures

Two cytokine panels were tested. The 13-plex Human Panel High Sensitivity quantified the following cytokines: IL-1beta, IL-2, IL-4, IL-5, IL-6, IL-8, IL-10, IL-12p70, IL-17a, GM-CSF, Interferon Gamma, MCP1 and TNFalpha. The designed 17-plex Human Panel quantified the following cytokines: MICA, MICB, IL-33, Fractalkine, RANTES, APRIL, REG3A, Perforin, CCL20, TSLP, IL-1 alpha, IL-23, IL-15, Granzyme B, CCL19, Granzyme A and VEGFA. Seven were excluded from further analysis: four of them were not detected at all in the supernatants (TSLP, IL-33, CCL19, IL 1 alpha), two due to an important batch effect (GM-CSF and VEGFA) and IL-15 because it was added in all the experiments.

#### Co-culture staining and microscopy acquisition

Samples in the selected wells were fixed 48 hours after the initiation of the co-culture and with a Paraformaldehyde (PFA) (Sigma-Aldrich) 4% solution and permeabilized with a Triton X-100 (Sigma-Aldrich) 0.1% solution. After a wash, a blocking solution of Bovine Serum Albumin (BSA) was then added to each well. The primary staining antibody mix was then added to each well composed of Mouse anti-E-Cadherin antibody (Abcam), Rat anti-CD3 antibody (Abcam) and Rabbit anti-Caspase 3 antibody (Abcam) at the concentration of 1/200 and incubated overnight at 4°C. After a wash, 250 μl of the secondary staining antibody mix was added to each well composed of Goat Anti-Mouse AF488 conjugated antibody (Abcam), Goat Anti-Rat AF555 conjugated antibody (Abcam) and Goat anti-Rabbit AF647 conjugated antibody (Abcam) and incubated for 2 hours at room temperature. Samples were then counterstained with a DAPI solution (1/50000 – Invitrogen). After a wash, a transparisation solution was added to each well: Rapiclear 1.47 (SunJin Lab).

Samples were imaged using a confocal spinning disk microscope (Nikon TiE) and data were processed using the Icy software.

### Quantification and statistical analysis

#### Microscopy images quantification

Images were quantified using the Icy software. A cut-off value was selected detecting the most specific staining. The same cut-off value for each staining was conserved across the distinct conditions. Areas of fluorescence were then extracted (**Supplementary Figure 2A**). The total volume of DAPI staining was considered as an estimation for the organoid volume. The volume of caspase 3 staining and the number of infiltrated T cells for each organoid were reported to the organoid volume to obtain an epithelial cell apoptosis and a T infiltrated cells rate for each structure. For each patient, the median value of all the data of organoid volume, cell apoptosis rate and T cells infiltrating the organoid for each condition was plotted.

#### Live imaging quantification

For live imaging acquisitions, all CD45+ cells were detected, counted (HK means cell detector plugin) and tracked for the 10 hours on all the z stacks (Spot tracking and Track manager plugin). The cell tracking was realized only for cells in contact with the organoid. The average speed and total displacement were calculated by Icy software (Motion profiler plugin of Track manager). The instant speed of each cell for each time point were also recovered (Instant speed plugin of Track manager) and analyzed with Rstudio software to calculate the arrest coefficient. This coefficient was defined as the percentage of time during which the cell was arrested (3 successive arrest time lapse) on all its trajectory.

### Statistical analysis

Descriptive statistics were calculated for all quantitative variables as medians with their respective interquartile ranges (IQRs). For phenotypic, microscopic and cytokine quantification data, variables data was also expressed as medians. Differences between conditions were evaluated with Mann-Whitney test for unpaired comparisons and Wilcoxon matched-pairs signed rank test when appropriate i.e when comparing experiments with patients in different conditions or timepoints. Spearman’s rank correlation test was used when necessary to evaluate the correlation between variables.

All statistical analyses were 2-tailed and a P value of <0.05 was considered as statistically significant. To perform these analyses, R software version 3.2.2 (R Foundation for Statistical Computing, Vienna, Austria) and GraphPad Prism (GraphPad Software, San Diego, CA) were used.

### Study approval

The ELYP cohort was approved by the French Ethic Committee - Hôpital Saint-Louis (CPP 2016-01-05 RBM) and declared to ClinicalTrials.gov (NCT02693340).

The REMIND study was approved by Agence Française de Sécurité Sanitaire et des Produits de Santé (AFSSAPS) (IDRCB: 2009-A00205-52) and the French Ethic Committee - Hôpital Saint-Louis (CPP 2009/17) and declared to ClinicalTrials.gov (NCT03458195).

The IMCO cohort was approved by the French Ethic Committee - Hôpital Saint-Louis (CPP 2016/45) and declared to ClinicalTrials.gov (NCT03015038).

All patients provided an informed written consent form.

## Data availability statement

The raw data supporting the conclusions of this article will be made available by the authors, without undue reservation.

## Ethics statement

The studies involving human participants were reviewed and approved by The ELYP cohort was approved by the French Ethic Committee - Hôpital Saint-Louis (CPP 2016-01-05 RBM) and declared to ClinicalTrials.gov (NCT02693340). The REMIND study was approved by Agence Française de Sécurité Sanitaire et des Produits de Santé (AFSSAPS) (IDRCB: 2009-A00205-52) and the French Ethic Committee - Hôpital Saint-Louis (CPP 2009/17) and declared to ClinicalTrials.gov (NCT03458195). The IMCO cohort was approved by the French Ethic Committee - Hôpital Saint-Louis (CPP 2016/45) and declared to ClinicalTrials.gov (NCT03015038). All patients provided an informed written consent form. The patients/participants provided their written informed consent to participate in this study.

## Author contributions

NH, LLB and MA: designed the experiments and interpreted the data. NH and SH: performed the co-culture experiments. JB and KP: performed statistical work. HB, VC, and DH: handled and processed patient samples. MB: managed the patient cohorts. MA, DH, JB, M-LT-M, J-MG, CB, HC and LM: included patients. J-MB, CB and MN: provided monoclonal antibodies and gave their scientific contribution to the study. NH, SH, LLB and MA wrote the manuscript. All authors contributed to the article and approved the submitted version.

## Funding

We thank Institut Roche and Genentech (MTA ID: OR-130059) for funding this work and providing etrolizumab and vedolizumab for the blockade experiments. We thank the Helmsley Charitable Trust, INSERM, and La Fondation pour la Recherche Médicale (FDM201806006069) for their financial support.

## Acknowledgments

We thank all members of the U1160 laboratory for their valuable help. We thank all the investigators and staff from the REMIND study group for the collection and data management of patients included in the study. We are grateful to the Technological Core Facility (Plateforme Technologique de l’IRSL) of the Institut de Recherche Saint-Louis and Institut Imagine for their continuous support in microscopy techniques. We also thank the Cytomorpholab at the Hôpital Saint-Louis, Unité de Thérapie Cellulaire and especially Manuel Thery and Benoît Viannay for their precious help including access to their microscopy platform and expertise. We finally thank Dr Dominique Cazals-Hatem, Nathalie Colnot from the Beaujon hospital pathology department that processed the samples paraffin blocks and performed the histological analysis of margins.

## Conflict of interest

MA received grant supports from Innate Pharma, Janssen, Takeda, Genentech/Roche, and honoraria for teaching activities or consultancy from Abbvie, Amgen, Biogen, Boehringer-Ingelheim, Bristol Myers Squibb, Celgene, Celltrion, Ferring, Genentech, Gilead, IQVIA, Janssen, Novartis, Pfizer, Roche, Takeda, Tillots. NH received honorarium for consultancy from Janssen and Takeda. M-LT-M received honoraria from Abbvie and Janssen. J-MG has been a speaker and/advisory board member for Abbvie, Amgen, Celltrion, Takeda, Janssen and Sanofi Genzyme. SH, JB, HB, KP, LC, MB, VC, BG, CG, DH, JB, HC, LM, CB, AT and LLB have no conflict of interest to declare. MN, JM are employed by Genentech and CB by Institut Roche.

The remaining authors declare that the research was conducted in the absence of any commercial or financial relationships that could be constructed as a potential conflict of interest.

## Publisher’s note

All claims expressed in this article are solely those of the authors and do not necessarily represent those of their affiliated organizations, or those of the publisher, the editors and the reviewers. Any product that may be evaluated in this article, or claim that may be made by its manufacturer, is not guaranteed or endorsed by the publisher.
