# Supplementary material for: Autologous organoid co-culture model reveals T cell-driven epithelial cell death in Crohn’s Disease
Source: Front Immunol. 2022 Nov 10;13:1008456. doi: 10.3389/fimmu.2022.1008456 (PMC9685428; doi:10.3389/fimmu.2022.1008456)
Supplement: Supplementary file 1 [file DataSheet_1.pdf]

ELYP Phenotype cohort	n = 85
Gender	
- Male	44 (52%)
- Female	41 (48%)
IBD Type and Location	
- Colonic Crohn’s Disease	28 (33%)
- Ileal Crohn's Disease	12 (14%)
- Ulcerative Colitis	39 (46%)
- Pouchitis	6 (7%)
Treatment Initiated	
-Anti TNF	49 (58%)
-Vedolizumab	16 (19%)
-Ustekinumab	20 (23%)
Previous Surgery	27 (31%)
Smoking	
- No smokers	68 (79%)
- Active smokers	12 (14%)
- NA	5 (7%)
Extra intestinal manifestations	
- Articular	15 (17%)
- Cutaneous	4 (5%)
- Ocular	3 (3%)
Previous exposure to	
- Azathioprine	65 (76%)
- Methotrexate	29 (34%)
Endoscopic response at the end of the study	
- No Response	24 (28%)
- Response	26 (31%)
- Remission	17 (20%)
- No endoscopy (Study exit or lost to follow-up)	18 (21%)
Phenotype at inclusion	
- Yes	81 (95%)
- No	4 (5%)
Phenotype at the end of the study	45 (53%)
- Yes	40 (47%)
- No	
