# Supplementary material for: Autologous organoid co-culture model reveals T cell-driven epithelial cell death in Crohn’s Disease
Source: Front Immunol. 2022 Nov 10;13:1008456. doi: 10.3389/fimmu.2022.1008456 (PMC9685428; doi:10.3389/fimmu.2022.1008456)
Supplement: Supplementary file 2 [file DataSheet_2.pdf]

REMIND Phenotype cohort	n=72
Gender	
- Male	34 (47%)
- Female	38 (53%)
Median Age, yr (IQR)	32.6 (26.8-45.0)
Age at CD Diagnosis	
-<16 years (A1)	11 (15%)
-17-40 years (A2)	53 (74%)
->40 years (A3)	8 (11%)
Median disease duration, yr (IQR)	10.4 (3.85-19.4)
Smoking	
- Non smokers	27 (38%)
- Active smokers at surgery	31 (43%)
- Ex smokers	14 (19%)
Previous intestinal resection	
-Yes	22 (31%)
-No	50 (69%)
Number of previous resection(s)	
- 0	50 (69%)
- 1	14 (19%)
- 2	5 (7%)
- 3	3 (5%)
Surgical indication	
-Stricturing complication	38 (53%)
-Penetrating complication	33 (46%)
-Failure of drug therapy	1 (1%)
Disease location (Montreal classification)	
- Ileal (L1)	43 (60%)
- Ileocolonic (L3)	29 (40%)
- Anoperineal lesions	13 (18%)
Extradigestive symptoms	
-Joint manifestations	8 (11%)
-Skin manifestations	3 (4%)
-Eye manifestations	0
Previous exposure to anti-TNF therapy	44 (61%)
Previous exposure to thiopurines	51 (71%)
Inflamed margin of resection	9 (13%)
