# Supplementary figures and images for: Autologous organoid co-culture model reveals T cell-driven epithelial cell death in Crohn’s Disease
Source: Front Immunol. 2022 Nov 10;13:1008456. doi: 10.3389/fimmu.2022.1008456 (PMC9685428; doi:10.3389/fimmu.2022.1008456)

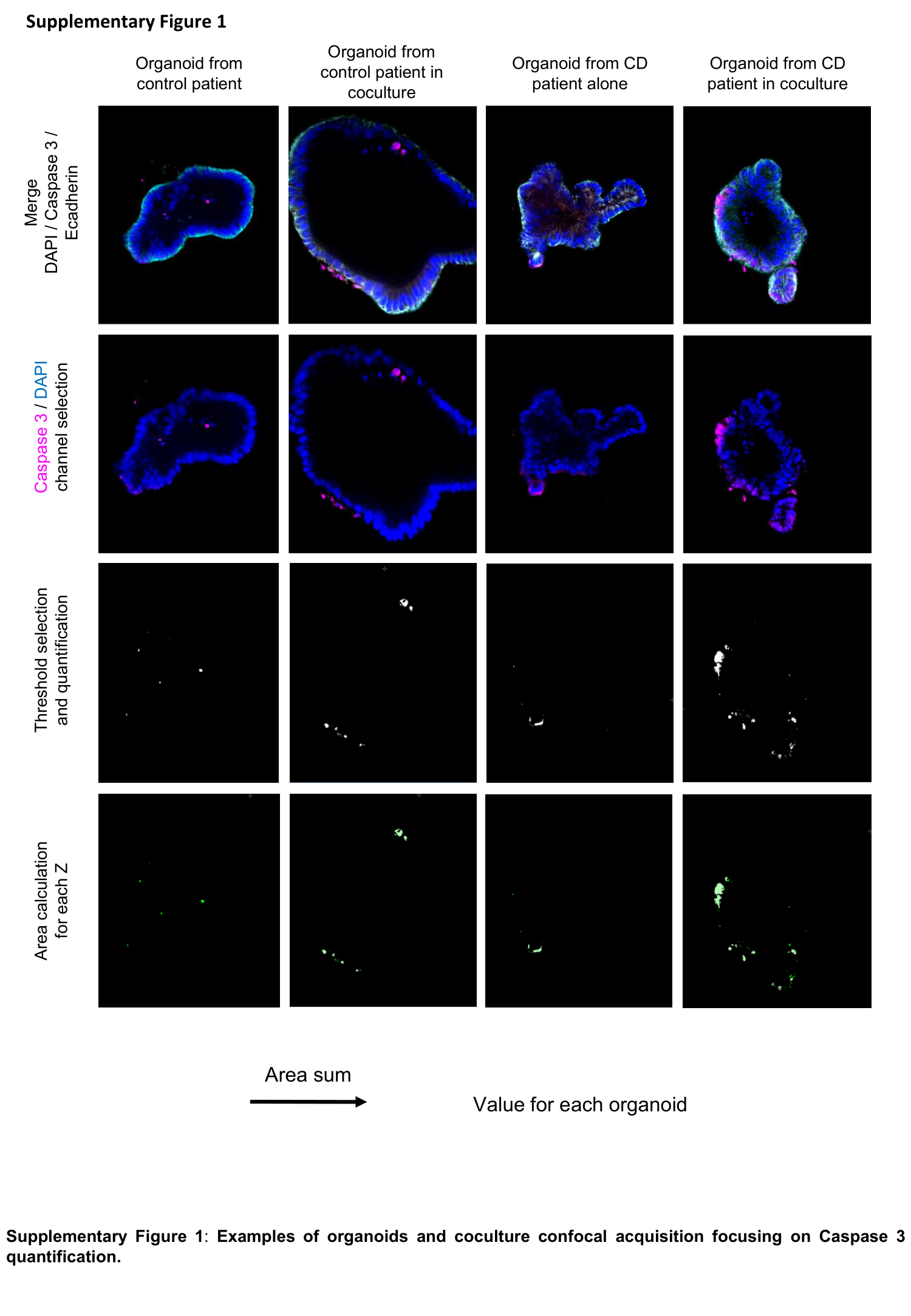

Supplement: Supplementary file 3 [file Image_1.tiff]

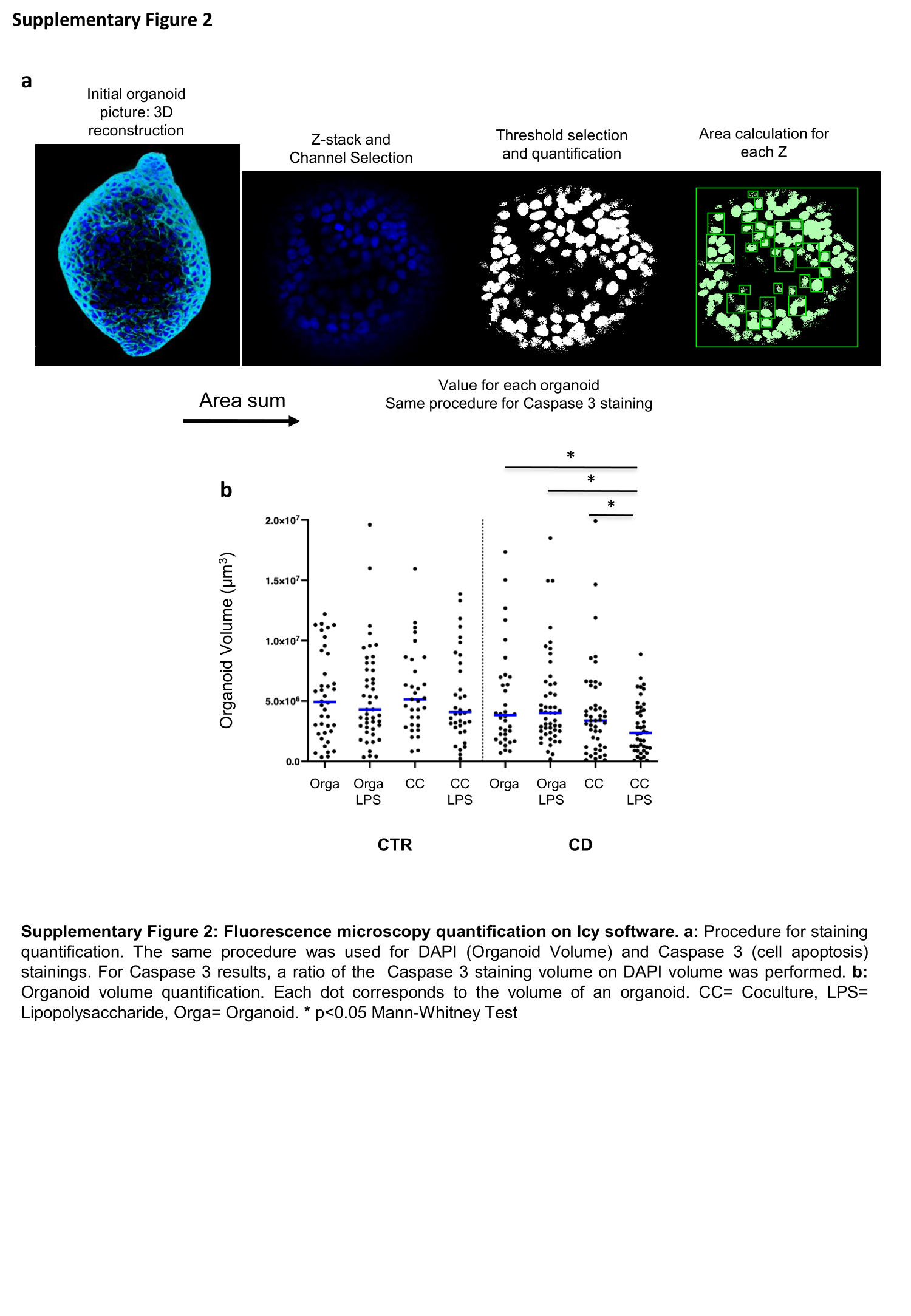

Supplement: Supplementary file 4 [file Image_2.tiff]

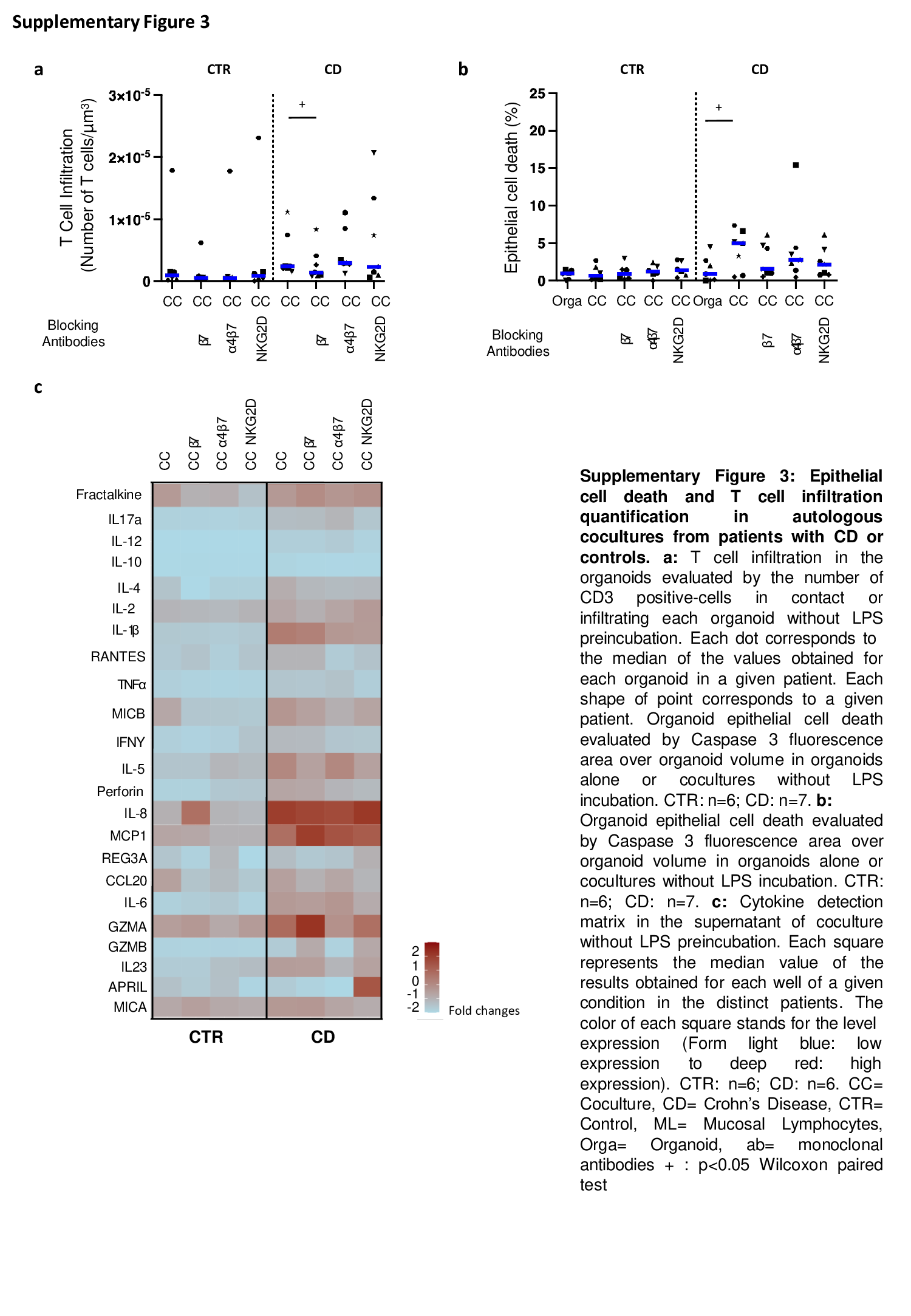

Supplement: Supplementary file 5 [file Image_3.tiff]

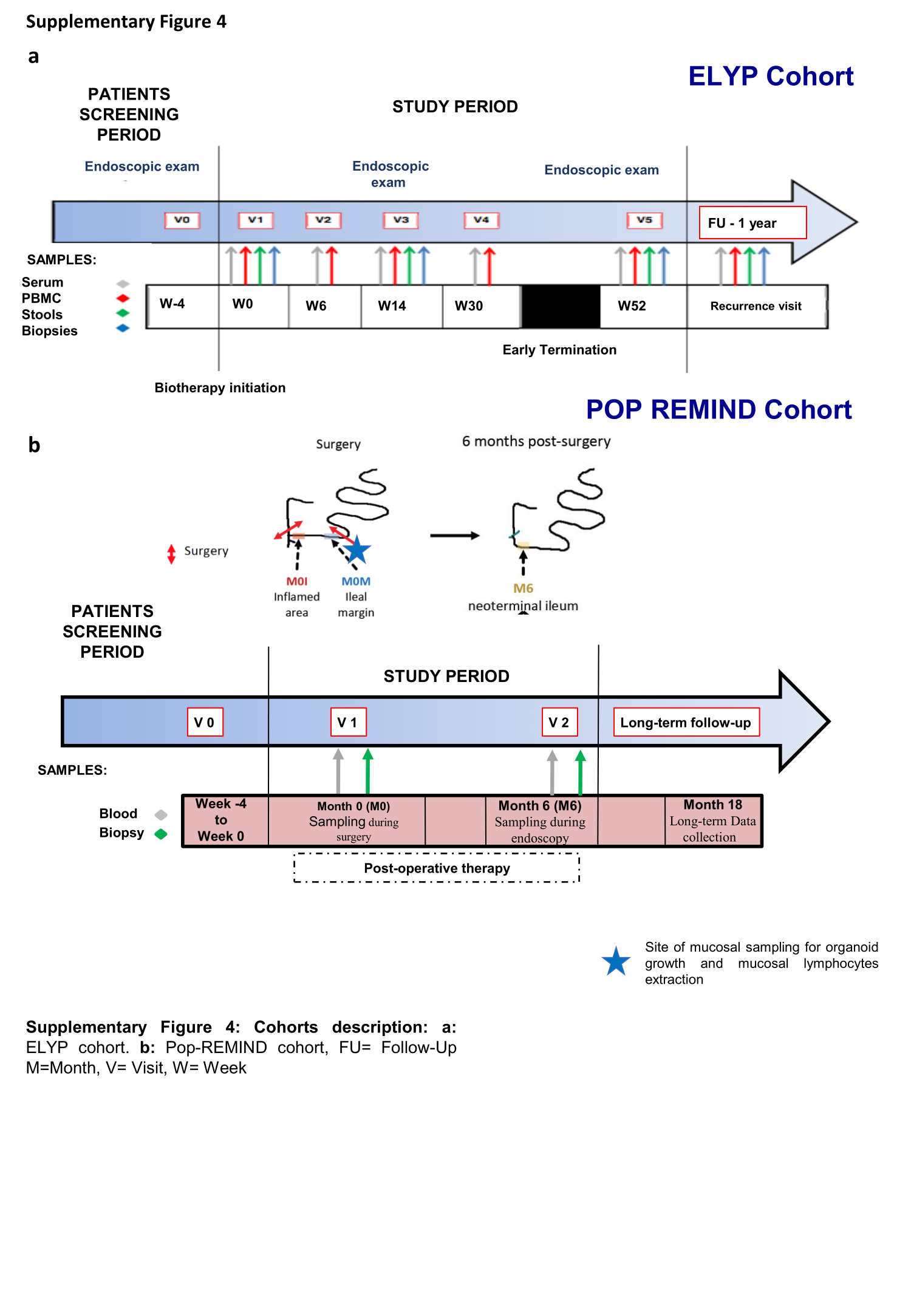

Supplement: Supplementary file 6 [file Image_4.tiff]

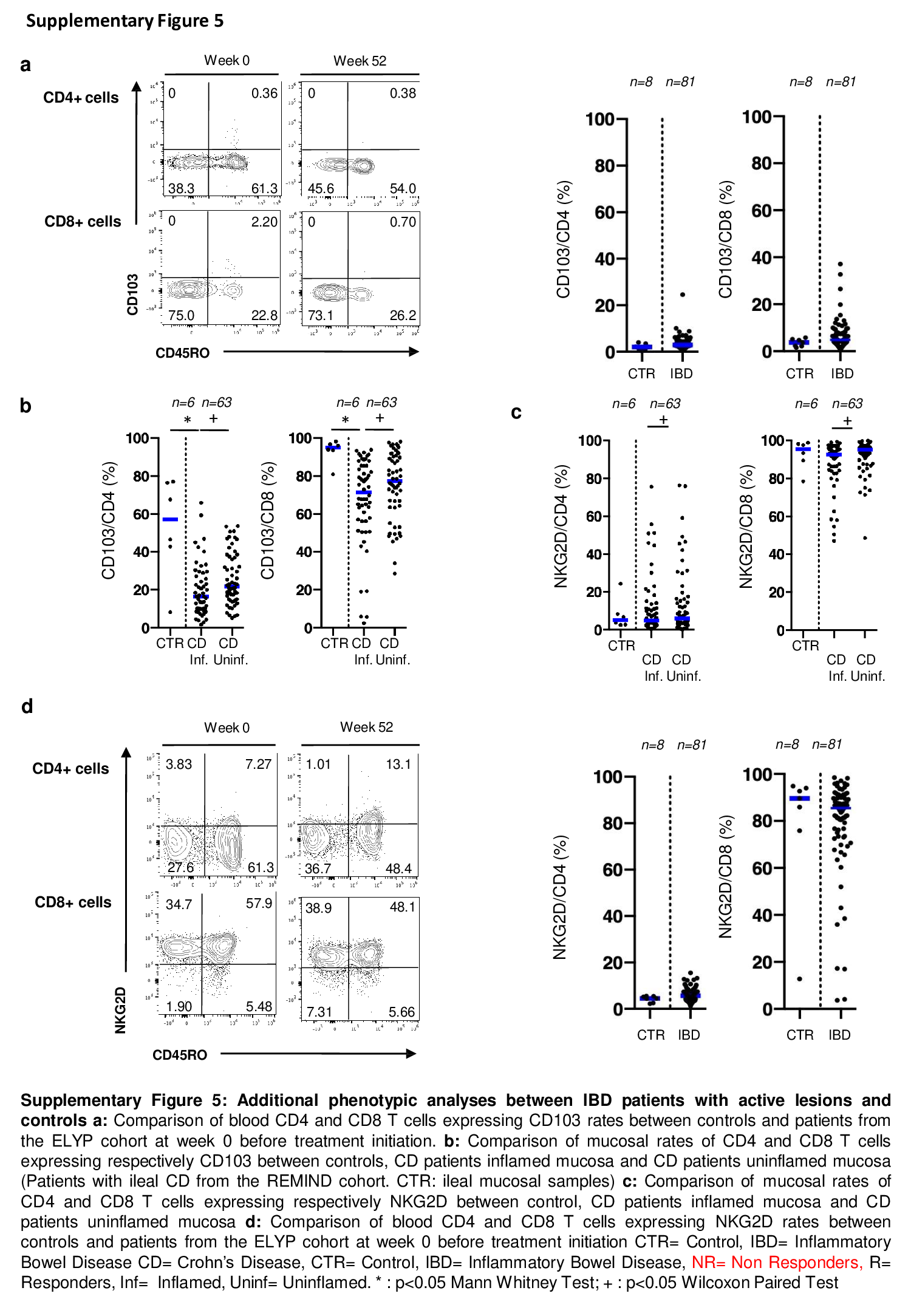

Supplement: Supplementary file 7 [file Image_5.tiff]

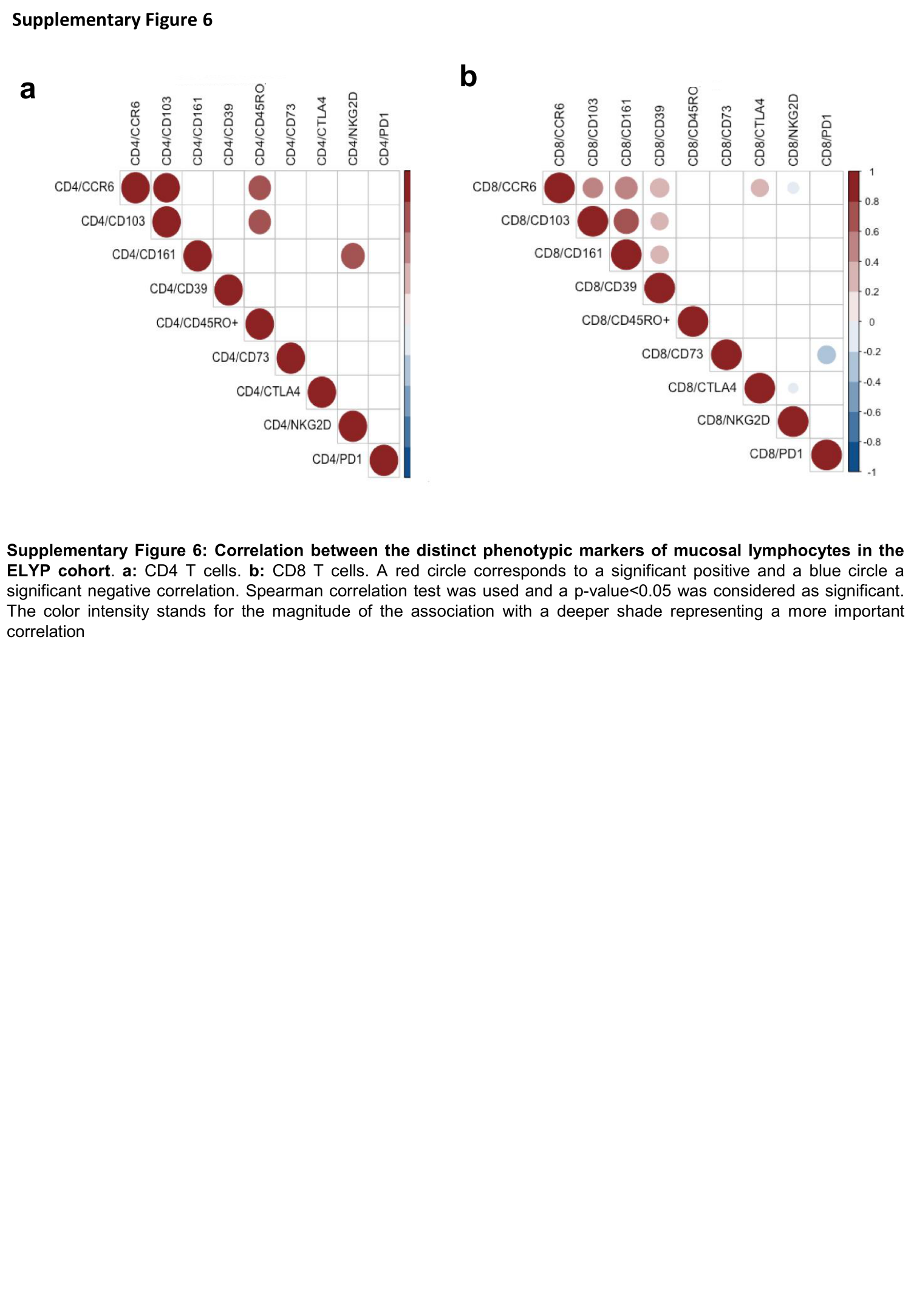

Supplement: Supplementary file 8 [file Image_6.tiff]

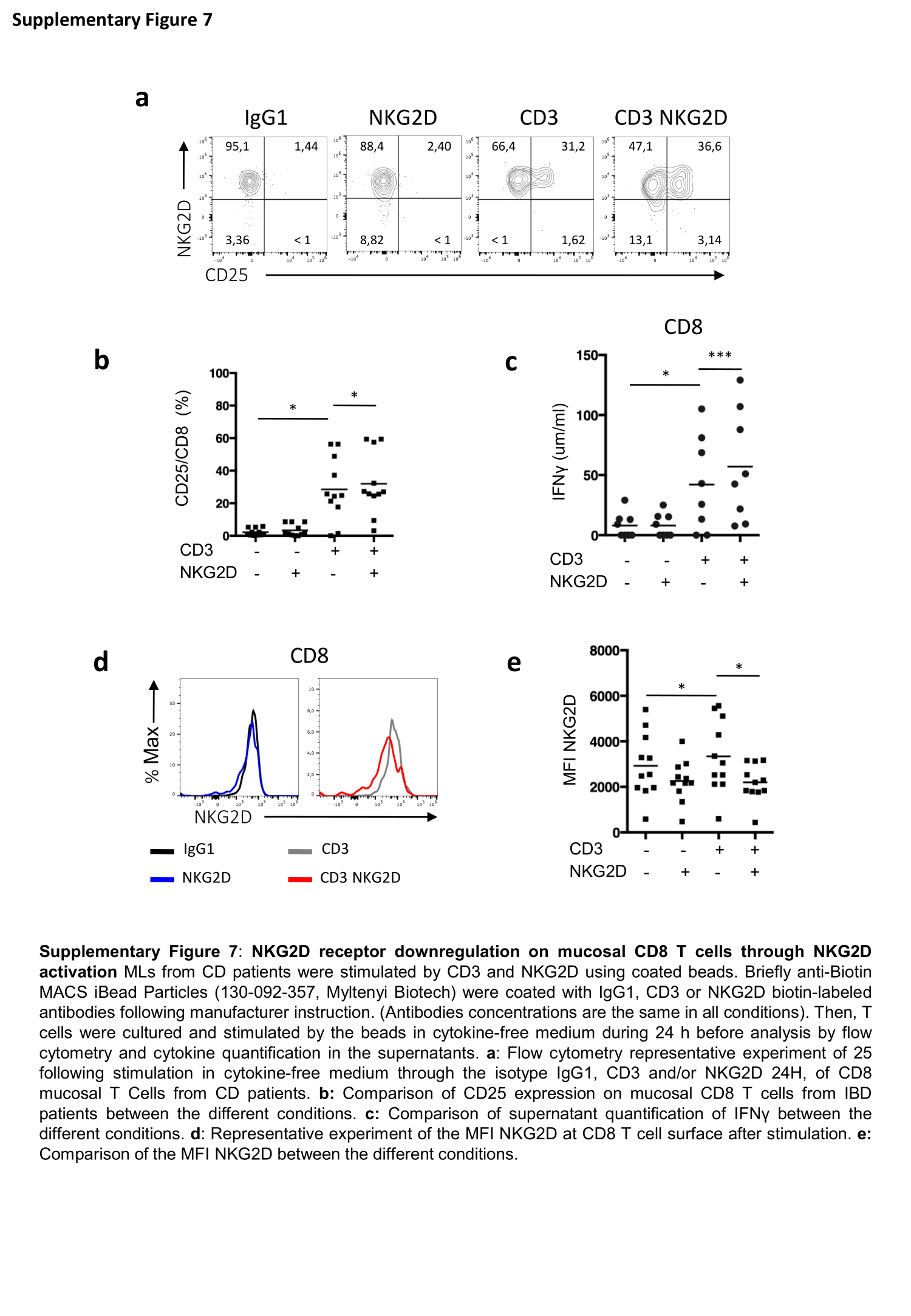

Supplement: Supplementary file 9 [file Image_7.tiff]
